# Overexpression of myocardin induces partial transdifferentiation of human‐induced pluripotent stem cell‐derived mesenchymal stem cells into cardiomyocytes

**DOI:** 10.1002/phy2.237

**Published:** 2014-02-25

**Authors:** Jiao Zhang, Jenny Chung‐Yee Ho, Yau‐Chi Chan, Qizhou Lian, Chung‐Wah Siu, Hung‐Fat Tse

**Affiliations:** 1 Division of Cardiology, Department of Medicine, Queen Mary Hospital, The University of Hong Kong, Hong Kong SAR, China; 2 Research Centre of Heart, Brain, Hormone & Healthy Aging, Li Ka Shing Faculty of Medicine, The University of Hong Kong, Hong Kong SAR, China; 3 Department of Ophthalmology, Li Ka Shing Faculty of Medicine, The University of Hong Kong, Hong Kong SAR, China

**Keywords:** Cardiomyocytes, mesenchymal stem cell, myocardin, transdifferentiation

## Abstract

Mesenchymal stem cells (MSCs) derived from human‐induced pluripotent stem cells (iPSCs) show superior proliferative capacity and therapeutic potential than those derived from bone marrow (BM). Ectopic expression of myocardin further improved the therapeutic potential of BM‐MSCs in a mouse model of myocardial infarction. The aim was of this study was to assess whether forced myocardin expression in iPSC‐MSCs could further enhance their transdifferentiation to cardiomyocytes and improve their electrophysiological properties for cardiac regeneration. Myocardin was overexpressed in iPSC‐MSCs using viral vectors (adenovirus or lentivirus). The expression of smooth muscle cell and cardiomyocyte markers, and ion channel genes was examined by reverse transcription‐polymerase chain reaction (RT‐PCR), immunofluorescence staining and patch clamp. The conduction velocity of the neonatal rat ventricular cardiomyocytes cocultured with iPSC‐MSC monolayer was measured by multielectrode arrays recording plate. Myocardin induced the expression of *α*‐MHC, GATA4, *α*‐actinin, cardiac MHC, MYH11, calponin, and SM *α*‐actin, but not cTnT, *β*‐MHC, and MLC2v in iPSC‐MSCs. Overexpression of myocardin in iPSC‐MSC enhanced the expression of SCN9A and CACNA1C, but reduced that of KCa3.1 and Kir2.2 in iPSC‐MSCs. Moreover, BK_Ca_, I_Kir_, I_Cl_, I_to_ and I_Na.TTX_ were detected in iPSC‐MSC with myocardin overexpression; while only BK_Ca_, I_Kir_, I_Cl_, IK_DR_, and IK_Ca_ were noted in iPSC‐MSC transfected with green florescence protein. Furthermore, the conduction velocity of iPSC‐MSC was significantly increased after myocardin overexpression. Overexpression of myocardin in iPSC‐MSCs resulted in partial transdifferentiation into cardiomyocytes phenotype and improved the electrical conduction during integration with mature cardiomyocytes.

## Abstract (precis)

Forced myocardin expression in human‐induced pluripotent stem cell (hiPSC)‐derived mesenchymal stem cells lead to partial transdifferentiation into cardiomyocytes and smooth muscle cells phenotypes through modification in ion channel expression profile and electrical conduction velocity.

## Introduction

Myocardin was identified as a transcription coactivator of serum response factor in mouse (Wang et al. 2001). The human myocardin gene expresses three alternative spliced mRNA variants (Torrado et al. 2003) with the longest isoform predominantly found in the heart (Du et al. 2003). Myocardin draws intensive interests because it activates the cardiac gene expression and influences heart function. The knockdown of the myocardin gene reduces the expression of cardiac markers in *Xenopus* and chick embryos (Wang et al. 2001; Small et al. 2005; Chen et al. 2008). Myocardin‐deficient adult mouse exhibits early onset of heart failure, dilated cardiomyopathy, and premature death within 1 week (Huang 2009) suggesting that myocardin is essential for the development of ventricular cardiomyocytes (Hoofnagle et al. 2011). Overexpression of myocardin induced the expression of various cardiac‐specific markers, such as cardiac troponin T (cTnT), MLC2a, MLC2v, *α*‐MHC, *β*‐MHC, GATA4, and sarco/endoplasmic reticulum Ca^2+^‐ATPase 2a (SERCA2a) in human bone marrow (BM)‐mesenchymal stem cells (MSCs) (van Tuyn et al. 2005). In addition, myocardin regulates the expression of multiple smooth muscle cell (SMC) differentiation‐related genes, including the smooth muscle (SM) *α*‐actin, SM22*α*, and SM‐calponin (Du et al. 2003; van Tuyn 2003; Wang and Wang 2003; Yoshida et al. 2003; van Tuyn et al. 2005).

Furthermore, overexpression of myocardin further improved the therapeutic potential of BM‐MSCs in a mouse model of myocardial infarction (Grauss et al. 2008), though the underlying mechanism remains unclear. Our recent studies have suggested that MSCs derived from human‐induced pluripotent stem cells (iPSCs) have more potent proliferative capacity and exhibit better therapeutic efficacy in tissue regeneration than BM‐MSCs (Lian et al. 2010). The aim of this study was to elucidate the effects of myocardin overexpression on MSC transdifferentiation, electrophysiological properties, and cellular integration of human iPSC‐MSCs with rat cardiomyocytes by examining the expression of cardiac marker genes and ion channel genes, and measuring their electrical conduction velocity. In order to obtain a thorough examination on the effect of forced myocardin expression, both lentiviral and adenoviral transduction systems were used in this study. To mimic the situation of pulsed overexpression of myocardin at the initial stage of transdifferentation, the non‐integration approach of adenoviral system was used to provide a transient forced expression of myocardin. However, the lentiviral system was used to examine the effects of a sustainable myocardin overexpression via genome integration approach.

## Materials and Methods

### Cell culture

Human iPSC‐MSCs were derived from iPSCs (iMR90)‐4 (Lian et al. 2010). Human BM‐MSCs were purchased from the Lonza Walkersville, Inc. (Walkersville, MD). The cells were cultured as monolayers in DMEM (Thermo Scientific, Waltham, MA) supplemented with 10% fetal bovine serum (FBS) (Invitrogen, Carlsbad, CA), 5 ng/mL bFGF (Invitrogen), and 5 ng/mL EGF (Peprotech, Hamburg, Germany).

### Quantitative real‐time polymerase chain reactions

Total RNA was extracted from the human BM‐MSCs, human iPSC‐MSCs, human myocardin‐overexpressing iPSC‐MSCs, GFP‐overexpressing iPSC‐MSCs, or cardiomyocytes derived from the human iPSCs ([iMR90]‐4) (Lee et al. 2011), and it underwent RT‐PCR with various primers (Table 1) as detailed in our previous work (Zhang et al. 2012).

**Table 1.** Oligonucleotide sequences of primers used for RT‐PCR.

Gene name	Accession no.	Forward primer (5′–3′)	Reverse primer (5′–3′)	Product size (bp)
Myocardin	NM_001146312	TTCAGAGGTAACACAGCCTCC	TGATCCTCTCTAGCGTCTGCT	132
GAPDH	NM_002046	CCATCTTCCAGGAGCGAG	GCAGGAGGCATTGCTGAT	233
SERCA2*α*	NM_001110140	AAGCTATGGGAGTGGTGGTG	GCAATGCAAATGAGGGAGAT	138
*α*‐MHC	GI 191623	GATGCCCAGATGGCTGACTT	GGTCAGCATGGCCATGTCCT	275
*β*‐MHC	NM_080728.2	GCCAACACCAACCTGTCCAAGTTC	TGCAAAGGCTCCAGGTCTGAGGGC	203
cTnT	NM_000364	AGCATCTATAACTTGGAGGCAGAG	TGGAGACTTTCTGGTTATCGTTG	112
GATA4	NM_008092	TCTCCCAGGAACATCAAAACC	GTGTGAAGGGGTGAAAAGG	125
Mef2c	NM_001131005	ATCTGCCCTCAGTCAGTTGG	AGAAGGCAGGGAGAGATTTGA	73
Mlc2v	NM_010861	GACCCAGATCCAGGAGTTCA	AATTGGACCTGGAGCCTCTT	163
Cx40	NM_005266	CTGGGCTGGAAGAAGATCAG	TGTGCAGCTCTGGACTATGC	102
Cx43	NM_000165	ATGAGCAGTCTGCCTTTCGT	TCTGCTTCAAGTGCATGTCC	249
Cx45	NM_005497	AGCAGACAAGAAGGCAGCTC	TTAGGTTTGGGTTGGCTCTG	165
SM22	AB209555	AACAGCCTGTACCCTGATGG	CGGTAGTGCCCATCATTCTT	239
MYH11	NM_001040113	GGAGGATGAGATCCTGGTCA	TTAGCCGCACTTCCAGTTCT	182
KCNH1 (a.k.a. EAG1 or Kv10.1)	NM_172362	TGGATTTTGCAAGCTGTCTG	GAGTCTTTGGTGCCTCTTGC	476
Clcn3	NM_173872	CATAGGTCAAGCAGAGGGTC	TATTTCCGCAGCAACAGG	293
KCa1.1	U11058	ACAACATCTCCCCCAACC	TCATCACCTTCTTTCCAATTC	310
KCa3.1	NM_002250	CGGGAACAAGTGAACTCCAT	ACTGGGGAAAGTAGCCTGGT	239
CACNA1C	NM_199460	AACATCAACAACGCCAACAA	AGGGCAGGACTGTCTTCTGA	574
Kir2.1	NM_000891	AACAGGGAGGTGTGGACAAG	TAACCTGCTCTAGGGCTCCA	261
Kir2.2	NM_021012	GAGGCTATCACAGGCTCAGG	CCCCAAGTTAAAAACCAGCA	183
Kir2.3	NM_152868	GCTTTGAGCCTGTGGTCTTC	TTGGCTCTGTCCTGAGTGTG	480
Kv1.4	NM_002233	ACGAGGGCTTTGTGAGAGAA	CACGATGAAGAAGGGGTCAT	308
Kv4.2	NM_012281	GCTTGTCATCAATCCCCTTG	TCCAGTATCTGGGCTTTTCC	102
Kv4.3	NM_172198	ACGGAGACATGGTGCCTAAG	CCCTGCGTTTATCAGCTCTC	153
SCN9A	NM_002977	GCTCCGAGTCTTCAAGTTGG	GGTTGTTTGCATCAGGGTCT	446

### Electrophysiology

The expression of ion channels in iPSC‐MSC_LV_^myo^ and iPSC‐MSC_LV_^GFP^ was examined by patch clamp techniques as detailed in our previous work (Zhang et al. 2012). In brief, the trypsinized cells were first seeded onto the glass coverslips in a four‐well plate. After a 30–45 min recovery period in the incubator, the cells were transferred into Tyrode's solution for patch clamp experiments. Using a HEKA EPC‐10 patch clamp amplifier and the PULSE v8.77 software (HEKA Instruments Inc., Southboro, MA), standard whole‐cell patch clamp recordings were performed at room temperature. The action potentials were recorded under the current (C)‐clamp at 37°C. Then, 1.5 mm thin‐walled borosilicate glass tubing (1.2 mm outer diameter, 0.69 mm internal diameter) (Sutter Instrument Co., Novato, CA) were used to prepare pipettes using a P‐97 Flaming/Brown micropipette puller (Sutter Instrument Co. Novato, CA). The typical pipette resistances were 3–4 MΩ when filled with internal pipette solution. After membrane rupture, the resting membrane potential was recorded under C‐clamp without a current input. The recorded membrane potentials were corrected for a liquid junction potential of +15.6 mV under room temperature, and +15.9 mV under 37°C. During the action potential recordings, the cells were held at 0 pA with 0.1–1 nA stimulation for 5 msec to elicit a response.

### Adenovirus preparation

The human myocardin gene (NM_001146312.1) was first amplified via PCR from human cardiac cDNA and ligated into the pCR2.1‐TOPO shuttle vector (Invitrogen, Carlsbad, CA). The ORF was subsequently subcloned into pAD‐shuttle‐IRES‐GFP (Stratagene, La Jolla, CA) for adenovirus production. Myocardin‐ and GFP‐expressing adenoviruses were produced using the AdEasy™ XL Adenoviral Vector System (Stratagene, La Jolla, CA). The generated adenoviruses were purified using an Adeno Mini Purification ViraKit (Virapur LLC, SD, CA) before iPSC‐MSCs transduction.

### Lentivirus production and transduction

Myocardin or GFP gene was inserted into lentivirus expression vector pSIN‐EF2‐Pur (Addgene, MA), and cotransfected with pCMV‐dR8.91 and pMD2G plasmid into 293FT cells (Invitrogen, Carlsbad, CA) using Lipofectamine 2000. Lentivirus were collected 3 days post transfection, filtered (0.45 *μ*m), and used to transduce iPSC‐MSCs with polybrene (Millipore, Billerica, MA). The positively transduced cells were selected using 0.5 *μ*g/mL puromycin‐containing medium after 3 days post transduction.

### Immunofluorescence staining

Cells seeded onto glass coverslips were washed with PBS, and fixed with 2% formalin for 30 min at room temperature. The cells were then incubated overnight with primary antibodies (1:200 dilutions in PBS) at 4°C. The primary antibodies included goat polyclonal anti‐myocardin (Santa Cruz Biotechnology Inc., Santa Cruz, CA), mouse antihuman CD29 (Stemgent, San Diego, CA), mouse monoclonal anti‐*α*‐actinin (sarcomeric) (Sigma‐Aldrich, Inc., St. Louis, MO), mouse anti‐myosin heavy chain (cardiac) (Upstate Biotechnology Inc., Waltham, MA), mouse monoclonal antihuman *α*‐actin (smooth muscle) (Dako, Copenhagen, Denmark), mouse monoclonal anti‐troponin T (cardiac) (Thermo Scientific), and rabbit monoclonal anti‐calponin‐1 (Millipore, Billerica, MA). After washing twice with PBS, the cells were incubated with the secondary antibodies for 30 min at 4°C. The secondary antibodies consisted of Alexa‐594 labeled rabbit anti‐goat IgG and Alexa‐647 labeled rabbit anti‐mouse IgG or Alexa‐594 labeled donkey anti‐goat IgG and Alexa‐647 labeled donkey anti‐rabbit IgG for myocardin and calponin costaining. After washing twice, the cells were mounted onto glass slides with mounting medium containing 4′,6‐diamidino‐2‐phenylindole dihydrochloride (DAPI) (Invitrogen). Immunocomplex was examined by fluorescent microscopy (Olympus IX81). To facilitate overlaying multicolored imaging, Alexa 647 was switched to a virtual color of green (512 nm) using Xcellence Pro version 1.1 software.

### Neonatal rat cardiomyocytes isolation and multielectrode arrays recording

In order to obtain viable cardiomyocyte to assess the electric conductance of the iPSC‐MSCs, cardiomyocyte were isolated from neonatal rat hearts. The animal study protocol conforms to the Guide for the Care and Use of Laboratory Animals published by the United States National Institutes of Health and was approved by the ethics committee of the University of Hong Kong (1851‐09). Hearts of neonatal Sprague‐Dawley rats (0–2 days old) were dissected and rinsed with modified Hanks' balanced salt solution (Invitrogen, Carlsbad, CA) (supplemented with 0.81 mmol/L MgSO_4_, 20 mmol/L HEPES, 100 U/mL penicillin and 100 *μ*g/mL streptomycin, pH = 7.5 by NaOH). The ventricles were minced on ice and dissociated with 0.2% Trypsin 250 (BD Biosciences, San Jose, CA). The dissociated cells were centrifuged down and resuspended in prewarmed neonatal rat ventricular cardiomyocytes (NRVM) culture medium containing DMEM supplemented with 5% FBS, 100 U/mL penicillin, 100 *μ*g/mL streptomycin, 1.5 mmol/L vitamin B_12_ (Sigma‐Aldrich), 10 mg/mL insulin (Sigma‐Aldrich), 10 mg/mL transferrin (Sigma‐Aldrich), and 0.1 mmol/L bromodeoxyuridine (Sigma‐Aldrich). The cells were then filtered (70 *μ*m), and preplated onto a 10‐cm plate for exactly 1 h at 37°C.

The freshly isolated NRVMs were harvested, and mixed with myocardin‐ or GFP‐overexpressing iPSC‐MSCs at a ratio of 4:1, and seeded onto gelatin‐coated multielectrode arrays (MEA) plates (Multi Channel Systems, Reutlingen, Germany) at approximately 10^5^ cells in total. The MEA plates used in this study contained 60 titanium nitride electrodes with a 200 *μ*m interelectrode distance and 30 *μ*m electrode diameter. The MEA plates combined with their supporting hardware allowed the simultaneous recording of extracellular electrical signals from all electrodes with one electrode as the ground. After allowing the NRVMs to settle down overnight, the electrical signals of the cell mixture were recorded and analyzed using the Cardio 2D+ software (Version 1.0.0) (Multi Channel Systems, Reutlingen, Germany).

### Statistical analysis

The numeric data are presented as mean ± SEM. The statistical significance of the differences between group means was evaluated using analysis of variance (ANOVA) with post hoc Bonferroni test as appropriate. Differences with *P *<**0.05 were considered statistically significant.

## Results

### Myocardin overexpression in human iPSC‐MSCs via lentivirus and adenovirus‐mediated transfer

**Figure 1.**
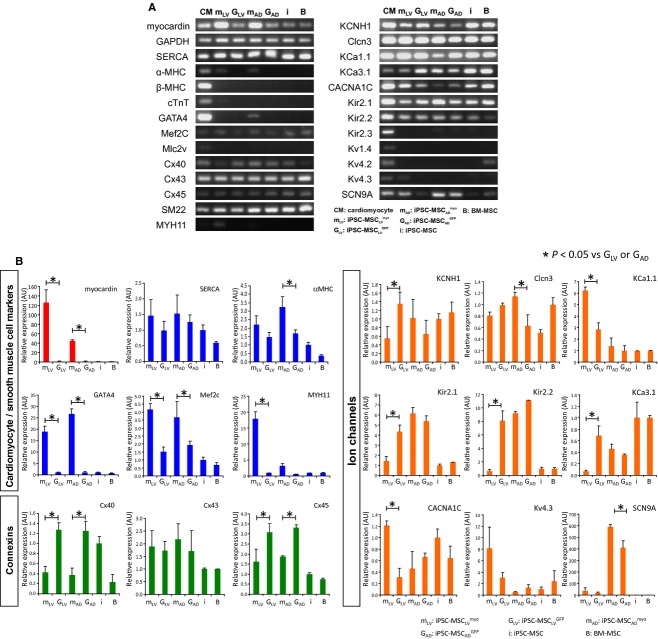
Gene expression analysis of myocardin expressing iPSC‐MSC. (A) Results of RT‐PCR for the detection of cardiomyocyte, smooth muscle cell marker genes, and ion channels genes expression. qPCR data are presented as relative fold change with reference to BM‐MSC. (B) Quantitative real‐time polymerase chain reaction (q‐PCR) gene expression analysis of myocardin expressing iPSC‐MSC. Relative expression cardiomyocyte, smooth muscle cell marker genes, connexins, and ion channels genes expression with reference to iPSC‐MSC are presented. No detectable expression MHC, cTnT, Mlc2V, Kir2.3, Kv1.4, and Kv4.2 were found in iPSC‐MSC. *P < 0.05 versus G_LV_ or G_AD_, respectively.

**Figure 2.**
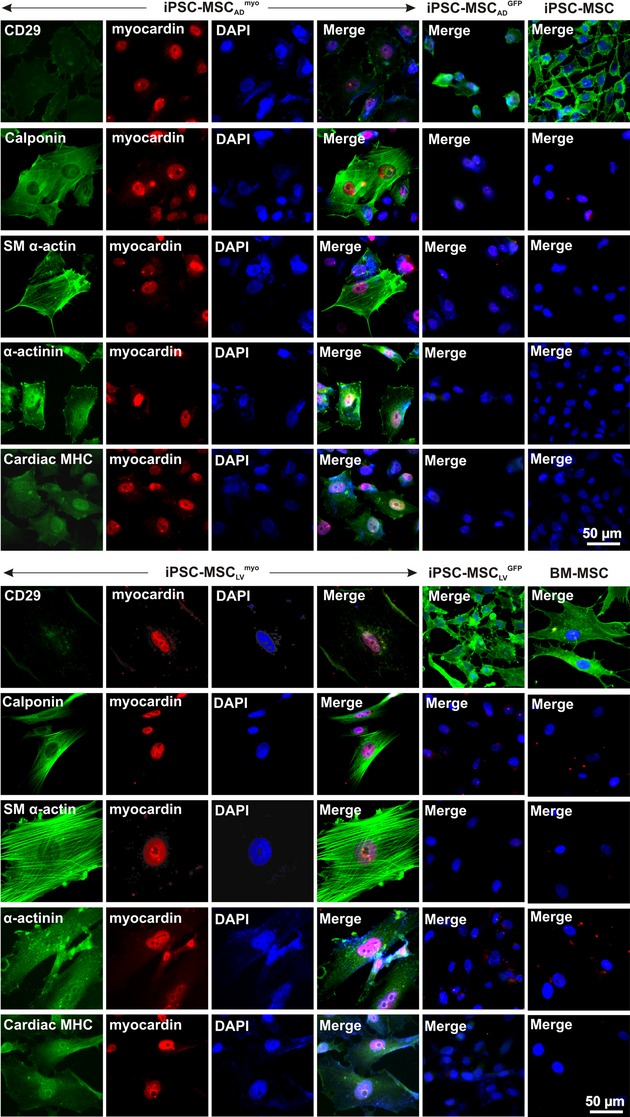
Immunofluorescence staining of myocardin with cardiomyocyte or smooth muscle cell marker genes as depicted in the figure. Adenovirus‐transduced samples were fixed 3 days post transduction, lentivirus transduced cells were fixed 12–14 days post transduction.

Positively transduced cells (i.e., iPSC‐MSC_LV_^myo^, iPSC‐MSC_LV_^GFP^, iPSC‐MSC_AD_^myo^, iPSC‐MSC_AD_^GFP^) were verified by RT‐PCR and immunofluorescence staining. As shown in Figure 1A, the mRNA expression level of myocardin was significantly higher in iPSC‐MSC_LV_^myo^ and iPSC‐MSC_AD_^myo^ than in their corresponding GFP controls. Quantitative RT‐PCR data revealed that the myocardin expression was 125‐ and 45‐fold higher after lentivirus and adenovirus‐mediated transfer, respectively, than the nontransduced cells (Fig. 1B). The myocardin protein was detected in the nucleus of iPSC‐MSC_LV_^myo^ and iPSC‐MSC_AD_^myo^, but not in their GFP controls (Fig. 2). Lentivirus transduced cells were selected with puromycin before further experiments.

### Overexpression of myocardin activated cardiomyocytes and smooth muscle cells marker genes of human iPSC‐MSCs

The expression of the cardiac marker genes, including *α*‐MHC and GATA4, was observed in myocardin‐transduced human iPSC‐MSCs, although lower than that of the cardiomyocyte control (Fig. 1). The expression level of SERCA, SM22, and connexin43 (Cx43) did not demonstrate obvious changes upon myocardin transduction. Mef2c was significantly upregulated upon myocardin transduction (2.7‐fold) while the expression of connexin40 (Cx40) and connexin45 (Cx45) were downregulated in iPSC‐MSC_LV_^myo^ and iPSC‐MSC_AD_^myo^ (all *P *<**0.05, Fig. 1B). Moreover, there was no detectable expression of cTnT, *β*‐MHC, and MLC2v in iPSC‐MSC_LV_^myo^ and iPSC‐MSC_AD_^myo^. On the other hand, iPSC‐MSC_LV_^myo^ activated the expression of the SMC marker gene MYH11. Figure 2 illustrates the positive staining for calponin, SM *α*‐actin, *α*‐actinin, and cardiac MHC, along with myocardin, in iPSC‐MSC_AD_^myo^ and iPSC‐MSC_LV_^myo^, but not in iPSC‐MSC_AD_^GFP^, iPSC‐MSC_LV_^GFP^, iPSC‐MSCs, or BM‐MSCs.

### Overexpression of myocardin‐modulated ion channel profile of human iPSC‐MSCs

According to the RT‐PCR results (Fig. 1), the expression of Kv4.3 (responsible for I_to_) (2.2‐fold), SCN9A (for I_Na.TTX_) (1.9‐fold), and CACNA1C (for I_Ca.L_) (3.9‐fold) were upregulated, whereas the expression of KCNH1 (for IK_DR_) (2.5‐fold), KCa3.1 (for IK_Ca_) (9.9‐fold), Kir2.1 and Kir2.2 (for I_Kir_) (3.1‐ and 13.1‐fold, respectively) were downregulated in iPSC‐MSC_LV_^myo^ compared with that in iPSC‐MSC_LV_^GFP^(all *P *<**0.05). No significant difference in the expression of Clcn3 (for I_Cl_) was observed between iPSC‐MSC_LV_^myo^ and iPSC‐MSC_LV_^GFP^.

**Figure 3.**
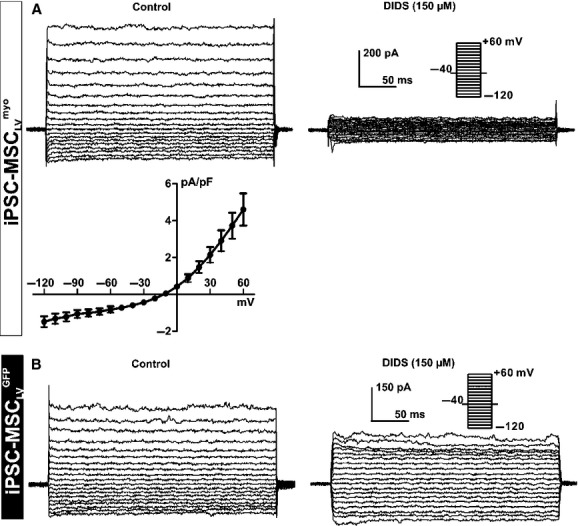
I_Cl_ in iPSC‐MSC_LV_^myo^ and iPSC‐MSC_LV_^GFP^. Voltage‐dependent current was inhibited by the Cl^−^ channel blocker DIDS (150 *μ*mol/L) in a representative iPSC‐MSC_LV_^myo^ (A) or iPSC‐MSC_LV_^GFP^ (B). Current was elicited by the protocol shown in the inset. I–V relationship of DIDS‐sensitive current obtained by subtracting the currents recorded before and after DIDS application in iPSC‐MSC_LV_^myo^ (*n* = 7) (A, lower panel)**.**

The functional expression of the ion channels was then further examined by patch clamp in iPSC‐MSC_LV_^myo^ (*n* = 282) and iPSC‐MSC_LV_^GFP^ (*n* = 101). Lentivirus‐mediated transduction combined with puromycin selection was adopted to ensure the successful transduction of myocardin or GFP in each cell for patch clamp. A current, likely I_Cl_ (Tao et al. 2007), was recorded in 26% of iPSC‐MSC_LV_^myo^ (72 of 282) and 3% of iPSC‐MSC_LV_^GFP^ (3 of 101) as shown in Figure 3. The identity of I_Cl_ was confirmed using a chloride channel blocker DIDS. The current was inhibited by DIDS (150 *μ*mol/L) in a representative iPSC‐MSC_LV_^myo^ (Fig. 3A) or iPSC‐MSC_LV_^GFP^ (Fig. 3B). Figure 3A lower panel illustrates the I–V relationship curve of the DIDS‐sensitive current in iPSC‐MSC_LV_^myo^ (*n* = 7), by subtracting currents recorded after DIDS administration from the control current accordingly.

**Figure 4.**
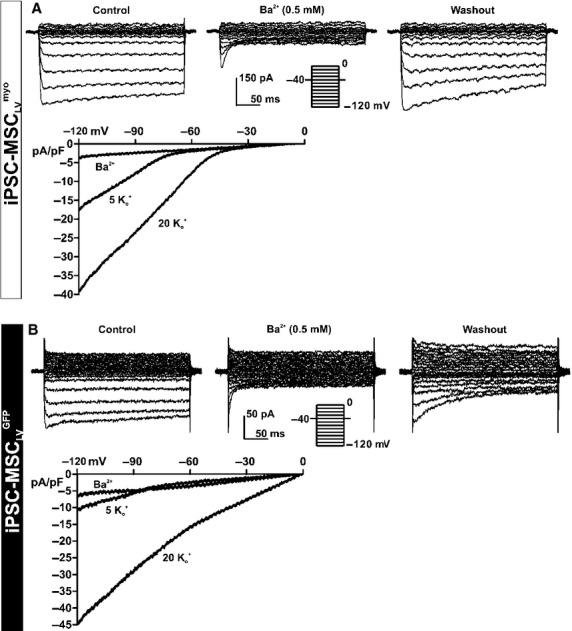
I_Kir_ in iPSC‐MSC_LV_^myo^ and iPSC‐MSC_LV_^GFP^. Voltage‐dependent currents, elicited with the inset protocol, were reversibly inhibited by BaCl_2_ (0.5 mmol/L) in a representative iPSC‐MSC_LV_^myo^ (A) or iPSC‐MSC_LV_^GFP^ (B), or with a 1.2‐sec ramp protocol (−120 to 0 mV from a holding potential of −40 mV) in another representative iPSC‐MSC_LV_^myo^ (A, lower panel) or iPSC‐MSC_LV_^GFP^ (B, lower panel) in the presence of 5 mmol/L K_o_^+^, 20 mmol/L K_o_^+^, or 0.5 mmol/L Ba^2+^ in bath solution.

This inward component exhibited properties similar to I_Kir_ (Deng et al. 2006) were observed in both iPSC‐MSC_LV_^myo^ (9%, 18 of 282) and iPSC‐MSC_LV_^GFP^ (12%, 11 of 101). The existence of I_Kir_ was determined using 0.5 mmol/L Ba^2+^, a specific blocker for I_Kir_. As displayed in Figure 4A and B, the inward rectifying K^+^ current was reversibly suppressed by 0.5 mmol/L BaCl_2_ in both iPSC‐MSC_LV_^myo^ and iPSC‐MSC_LV_^GFP^. The I–V relationships of I_Kir_, elicited with a 1.2 sec ramp protocol (from −120 to 0 mV) in a solution containing 5 mmol/L K^+^ (Tyrode's solution) or 20 mmol/L K^+^, and after application of 0.5 mmol/L Ba^2+^ in the bath solution are illustrated in a representative iPSC‐MSC_LV_^myo^ (Fig. 4A, lower panel) or iPSC‐MSC_LV_^GFP^ (Fig. 4B, lower panel). Similar results were observed in 12 iPSC‐MSC_LV_^myo^ and 4 iPSC‐MSC_LV_^GFP^.

**Figure 5.**
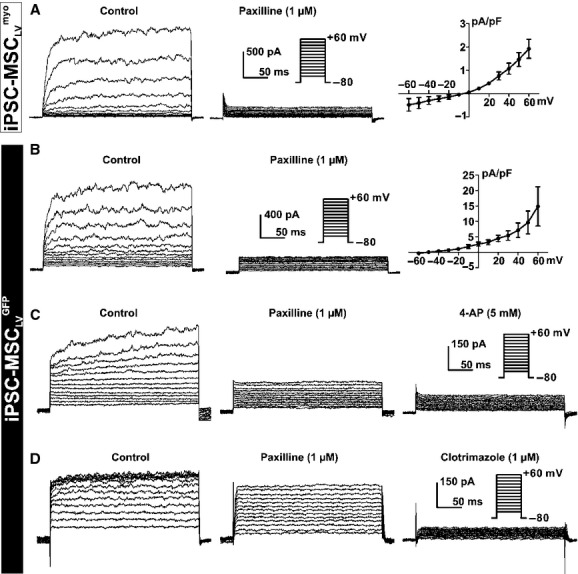
BK_Ca_ in iPSC‐MSC_LV_^myo^, BK_Ca_, IK_DR_, and IK_Ca_ in iPSC‐MSC_LV_^GFP^. Noisy currents were completely inhibited by paxilline (1 *μ*mol/L) in a representative iPSC‐MSC_LV_^myo^ (A) or iPSC‐MSC_LV_^GFP^ (B). I–V relationship of paxilline‐sensitive currents obtained by subtracting the currents before and after paxilline treatment accordingly in iPSC‐MSC_LV_^myo^ (*n* = 12) (A, right panel) and iPSC‐MSC_LV_^GFP^ (*n* = 5) (B, right panel). Voltage‐dependent currents in a representative iPSC‐MSC_LV_^GFP^ were partially inhibited by paxilline (1 *μ*mol/L) and completely abolished by 4‐AP (5 mmol/L) (*n* = 5) (C) or clotrimazole (1 *μ*mol/L) (*n* = 3) (D). Currents were elicited by the protocol shown in the inset.

An outward current, likely BK_Ca_ current (Li et al. 2005), was activated in both iPSC‐MSC_LV_^myo^ (68%, 191 of 282) and iPSC‐MSC_LV_^GFP^ (85%, 86 of 101) as shown in Figure 5A and B. The identity of BK_Ca_ was confirmed using the BK_Ca_ channel‐specific blocker paxilline (Fig. 5A and B). After administration of paxilline, the current activated at +60 mV, was significantly reduced from 2.9 ± 0.4 pA/pF to 1.0 ± 0.12 pA/pF in iPSC‐MSC_LV_^myo^ (*n* = 11, *P *<**0.001), and from 28.6 ± 8.4 pA/pF to 8.6 ± 1.9 pA/pF in iPSC‐MSC_LV_^GFP^ (*n* = 9, *P *<**0.05).

Two types of paxilline‐resistant currents were recorded in iPSC‐MSC_LV_^GFP^ but not in iPSC‐MSC_LV_^myo^. As shown in Figure 5C, a paxilline‐resistant current in a representative iPSC‐MSC_LV_^GFP^ was further inhibited by IK_DR_ blocker 4‐AP. Paxilline (1 *μ*mol/L) partially suppressed the membrane current at +60 mV from 19.2 ± 3.7 pA/pF to 9.2 ± 2.4 pA/pF. The remaining current was inhibited by 4‐AP (5 mmol/L) to 2.2 ± 1.5 pA/pF (*n* = 5, *P *<**0.05). The other type of current displayed properties of IK_Ca_ (Tao et al. 2007) was detected only in iPSC‐MSC_LV_^GFP^ (6%, 6 of 101). In Figure 5D, the outward current in a representative iPSC‐MSC_LV_^GFP^ was partially inhibited by paxilline (1 *μ*mol/L), with the remaining current completely blocked by the IK_Ca_ blocker clotrimazole (1 *μ*mol/L). Similar results were observed in five iPSC‐MSC_LV_^GFP^ cells.

**Figure 6.**
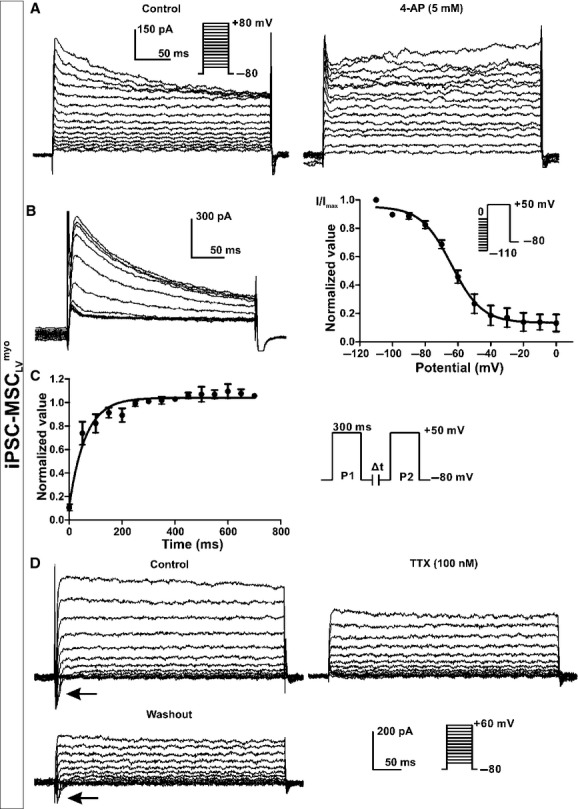
I_to_, and I_Na.TTX_ in iPSC‐MSC_LV_^myo^. (A) I_to_ currents in a representative iPSC‐MSC_LV_^myo^ were inhibited by 4‐AP (5 mmol/L). (B) Voltage‐dependent inactivation of I_to_ in a representative cell and normalized mean values (I/I_max_) under different voltages (*n* = 6). (C) The recovery curve of I_to_. P2 current was normalized by P1 current and plotted against the P1‐P2 interval (*n* = 5). (D) An inward current (arrow) recorded in a representative cell was reversibly inhibited by TTX (100 nmol/L) (*n* = 4). Currents were elicited by the protocol shown in the inset.

Two other types of currents were recorded in iPSC‐MSC_LV_^myo^ but not in iPSC‐MSC_LV_^GFP^. One was the transient outward K^+^ current I_to_ sensitive to 4‐AP (Li et al. 2005). I_to_ was detected in a small portion of iPSC‐MSC_LV_^myo^ (4%, 11 of 282) with a representative one shown in Figure 6A. The voltage‐dependent inactivation of I_to_ was assessed by the step potential at +50 mV after 1000 ms conditioning potentials from −110 to 0 mV (Fig. 6B). The I_to_ inactivation curve was obtained by plotting I/I_max_ of I_to_ (the peak value of I_to_ normalized to the maximum one) as a function of the conditioning potentials and fitting to the Boltzmann sigmoidal curve. The slope of the I_to_ inactivation curve was approximately 9.3 ± 1.7, and the half inactivated conditioning potential was approximately −64.0 ± 1.9 mV (*n* = 6). The time‐dependent recovery of I_to_ from inactivation was estimated with a paired‐pulse protocol (shown in the inset of Fig. 6C). The I_to_ recovery curve was fit to an exponential function‐one phase association with the time constant (*ζ*) 61.5 msec and half‐time 42.6 msec (*n* = 4).

The other current exclusively observed in iPSC‐MSC_LV_^myo^ was an inward current activated from approximately −30 to +60 mV and sensitive to tetrodotoxin (TTX). This phenomenon suggested the existence of I_Na.TTX_ (Li et al. 2005) in iPSC‐MSC_LV_^myo^ (4%, 12 of 282). Figure 6D demonstrates that TTX (100 nmol/L) reversibly inhibited the inward current, with limited effects on the outward current. Similar results were observed in four iPSC‐MSC_LV_^myo^ cells.

In summary, a similar functional ion channel profile (i.e., BK_Ca_, I_Kir_, I_Cl_, IK_DR_, and IK_Ca_) as in iPSC‐MSCs (Zhang et al. 2012) was observed in iPSC‐MSC_LV_^GFP^ (85%, 12%, 3%, 56%, and 6%). Two distinct ion channels, I_to_ and I_Na.TTX_ were recorded in iPSC‐MSC_LV_^myo^ (68%, 9%, 26%, 4%, and 4%).

Furthermore, iPSC‐MSC_LV_^myo^ had a RMP of −25.9 ± 0.7 mV, which was more depolarizing than that of iPSC‐MSC_LV_^GFP^ (−32.1 ± 1.6 mV) (*P *<**0.001). In addition, the cell size of iPSC‐MSC_LV_^myo^ was significantly increased upon myocardin transduction than iPSC‐MSC_LV_^GFP^ (*P *<**0.001), as reflected by their respective average membrane capacitance (69.3 ± 2.5 pF vs. 22.4 ± 1.7 pF).

### Myocardin overexpressed iPSC‐MSCs did not exhibit action potentials as mature cardiomyocytes

**Figure 7.**
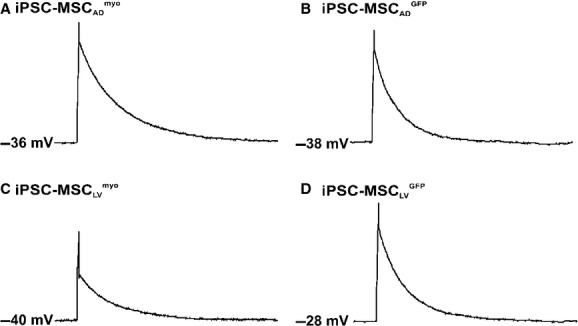
Action potentials in myocardin or GFP transduced iPSC‐MSCs. Action potentials shown in a representative human iPSC‐MSC_AD_^myo^ (A), iPSC‐MSC_AD_^GFP^ (B), iPSC‐MSC_LV_^myo^ (C), iPSC‐MSC_LV_^GFP^ (D), iPSC‐MSC_LV_^myo^ incubated in cardiomyocyte (CM) differentiation medium for 5 days (E) or its GFP control (F).

To assess whether overexpression of myocardin resulted in complete cardiomyocyte differentiation of iPSC‐MSCs, the action potential of iPSC‐MSC_LV_^myo^, iPSC‐MSC_LV_^myo^ incubated in cardiomyocyte differentiation medium, iPSC‐MSC_AD_^myo^, and their GFP controls (*n* = 11–23) were also characterized (Fig. 7). However, none of these cells exhibited action potentials as mature cardiomyocytes.

### Overexpression of myocardin significantly increased the electrical conduction velocity of human iPSC‐MSCs

**Figure 8.**
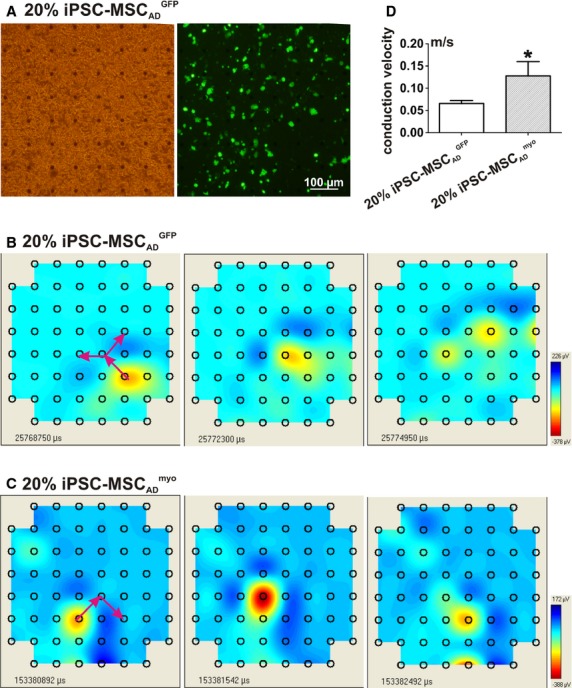
Cocultures of NRVMs and iPSC‐MSC. (A) NRVMs were cocultured with iPSC‐MSC_AD_^GFP^ on a MEA recording plate. The electrical signals transmitted through MEA plate were recorded in NRVMs cocultured with 20% iPSC‐MSC_AD_^GFP^ (B), or iPSC‐MSC_AD_^myo^ (C), with the average conduction velocity of 0.07 ± 0.01 m/sec and 0.13 ± 0.03 m/sec, respectively (D). *P < 0.05.

The effects of myocardin overexpression on the cellular integration and conductance changes of iPSC‐MSC with cardiomyocytes were determined by measuring the electrical conduction velocity in a monolayer coculture of iPSC‐MSC_AD_^myo^ or iPSC‐MSC_AD_^GFP^ with NRVMs on MEA recording plates (Fig. 8A). The electrical signals transmitted through the MEA plate were recorded as shown in a representative iPSC‐MSC_AD_^GFP^ (Fig. 8B) or iPSC‐MSC_AD_^myo^ (Fig. 8C), coculture system. The electrical conduction velocities of iPSC‐MSC_AD_^myo^ (*n* = 13; 0.13 ± 0.03 m/sec) were significantly faster than those of iPSC‐MSC_AD_^GFP^ (*n* = 15; 0.07 ± 0.01 m/sec) (Fig. 8D, *P* < 0.05).

## Discussion

Recent clinical studies have suggested that MSC is a potential cell source for cardiac regeneration in patients with myocardial infarction and heart failure (Siu and Tse 2012). The underlying mechanisms of the beneficial effect of MSC for cardiac regeneration likely mediated through multiple actions, including transdifferentiation into functional cardiomyocyte or fusion with native cardiomyocytes, paracrine effects, cell‐cell interactions, and integration with native cardiomyocytes (Williams and Hare 2011). Previous studies have shown that myocardin activates cardiac gene expression in BM‐MSCs (van Tuyn et al. 2005), and improved their therapeutic potential for myocardial infarction (Grauss et al. 2008).

This study is the first to investigate the effect of myocardin overexpression in human iPSC‐MSCs. Our previous studies have shown that iPSC‐MSCs exhibit higher proliferative potential and better therapeutic efficacy for tissue regeneration (Lian et al. 2010; Zhang et al. 2012). In this study, the myocardin overexpression in human iPSC‐MSCs resulted in partial transdifferentiation into cardiomyocyte phenotypes as reflected by the appearance of cardiac markers *α*‐MHC, GATA4, *α*‐actinin, and cardiac MHC, but not cardiac markers cTnT, *β*‐MHC, MLC2v, and cTnT protein. These findings were consistent with those observed in myocardin overexpression in BM‐MSC (van Tuyn et al. 2005; Grauss et al. 2008). Therefore, myocardin overexpression alone could not induce complete cardiomyocyte transdifferentiation from iPSC‐MSCs as observed in BM‐MSC. The incomplete transdifferentiation into mature cardiomyocyte phenotypes from myocardin‐transduced iPSC‐MSCs was further confirmed by the lack of mature action potentials recorded by patch clamping (data not shown). It is likely that those phenotypic changes of the MSC are due to secondary effect of multiple genes expression induced by myocardin rather than direct myocardin transcriptional activity to induce complete cardiac transdifferentiation. Moreover, our findings reveal several similar as well as different gene expression profiles related transient versus long‐term expression of myocardin using adenovirus and lentivirus transduction, respectively.

In this study, we characterized and compared the ion channel profiles of iPSC‐MSC_LV_^myo^ and iPSC‐MSC_LV_^GFP^ to assess the electrophysiological effects of myocardin overexpression on iPSC‐MSCs. RT‐PCR results revealed that iPSC‐MSC_LV_^myo^ has higher expression levels of Kv4.3 (responsible for I_to_), KCa1.1 (for BK_Ca_), SCN9A (for I_Na.TTX_), and CACNA1C (for I_Ca.L_), but lower expression levels of KCa3.1 (for IK_Ca_), KCNH1 (for IK_DR_), and Kir2.1 and Kir2.1 (for I_Kir_) than those of iPSC‐MSC_LV_^GFP^. Both iPSC‐MSC_LV_^myo^ and iPSC‐MSC_LV_^GFP^ had similar expression levels of Clcn3 (for I_Cl_). Patch clamp results showed that BK_Ca_, I_Kir_, I_Cl_, I_to_, and I_Na.TTX_ were expressed in iPSC‐MSC_LV_^myo^ (68%, 9%, 26%, 4%, and 4%), compared with BK_Ca_, I_Kir_, I_Cl_, IK_DR_ and IK_Ca_ in iPSC‐MSC_LV_^GFP^ (85%, 12%, 3%, 56%, and 6%). Overexpression of myocardin upregulated the functional expression of I_Cl_ channels and activated the functional expression of I_to_ and I_Na.TTX_ channels, but inhibited the functional expression of the IK_Ca_ IK_DR_ and I_Kir_ channels.

The expression of the human Clcn3 gene is mainly found in the skeletal muscle and brain (Borsani et al. 1995). The inactivation of the Clcn3 gene causes severe myocardial hypertrophy and heart failure in knockout mice (Xiong et al. 2010). Clcn3 encodes the I_Cl_ channel, which is volume‐sensitive and can be activated under hypotonic conditions, as shown in human gastric epithelial cells (Jin et al. 2003), mouse BM‐MSCs (Tao et al. 2007), and bovine epithelial cells (Wang et al. 2000). In this study, the size of the iPSC‐MSC_LV_^myo^ greatly expanded compared with iPSC‐MSC_LV_^GFP^, as reflected by the increase in the average membrane capacitance from 22.4 ± 1.7 pF to 69.3 ± 2.5 pF. The percentage of cells that displayed I_Cl_ currents increased from 3% to 26%. The activation of the volume‐sensitive I_Cl_ channel possibly resulted from the increase in the cell volume of iPSC‐MSC_LV_^myo^.

The I_to_ channel is encoded by Kv1.4, Kv4.2, and Kv4.3, which have been detected in the human heart (Gaborit et al. 2007). In this study, the expression of Kv4.3, but not Kv1.4 and Kv4.2, was found in iPSC‐MSC_LV_^myo^. Therefore, the I_to_ current recorded in iPSC‐MSC_LV_^myo^ is likely mediated by the expression of Kv4.3. The I_to_ current is one of the critical components of the action potential in mature ventricular cardiomyocytes (Amin et al. 2010). 4‐AP sensitive I_to_ current has been observed in atrial and ventricular cardiomyocytes (Li et al. 1995, 1998). In this study, I_to_ current has been detected in only 4% of iPSC‐MSC_LV_^myo^. These results suggest that overexpression of myocardin directs the transdifferentiation of iPSC‐MSCs to cardiomyocyte phenotypes, although at a very low efficiency.

In addition, myocardin can activate the expression of SMC marker genes in mouse ESCs (Du et al. 2003), fibroblast (Wang and Wang 2003), human BM‐MSCs and fibroblast (van Tuyn et al. 2005), and human ventricular scar fibroblast (van Tuyn 2003). In this study, myocardin induced the expression of SMC marker genes (i.e., SM22, MYH11, calponin, and SM *α*‐actin) in the iPSC‐MSCs. However, myocardin expression did not induce complete SMC transdifferentiation (Yoshida et al. 2004). Although the potential role of SMC transdifferentiation remains unclear, MSC‐derived SMC might enhance vasculogenesis.

The expression of SCN9A mRNA is significantly upregulated in iPSC‐MSC_LV_^myo^ compared with that in iPSC‐MSC_LV_^GFP^ or non‐transduced cells. SCN9A might be responsible for the I_Na.TTX_ current recorded in iPSC‐MSC_LV_^myo^ (4%). The I_Na.TTX_ current has been observed in the human BM‐MSCs (Li et al. 2005) and cardiac fibroblasts (Li et al. 2009). However, no TTX‐resistant sodium current encoded by SCN5A has been recorded in the iPSC‐MSC_LV_^myo^. The expression level of SCN5A is much higher than that of SCN9A in the human heart (Gaborit et al. 2007). Furthermore, other important ion currents, such as I_Ca.L_and I_f_ that compose the action potential of cardiomyocytes (Amin et al. 2010) have not been detected in iPSC‐MSC_LV_^myo^. These data again suggest that myocardin induces incomplete cardiomyocyte transdifferentiation from iPSC‐MSCs.

In addition to the induction of the functional expression of the I_to_ and I_Na.TTX_ channels, forced myocardin expression interferes with the expression of the IK_Ca_ and the ether à go‐go 1 potassium (responsible for IK_DR_) channels. The occurrence of the IK_Ca_ current (possibly encoded by the KCa3.1) in iPSC‐MSC_LV_^GFP^ is approximately 6%, which is comparable with those in iPSC‐MSCs (5%, (Zhang et al. 2012)). The absence of the IK_Ca_ current in iPSC‐MSC_LV_^myo^ might have resulted from the decreased expression of KCa3.1. As we have shown previously, the ether à go‐go 1 potassium channel is critical in the regulation of the proliferation of iPSC‐MSCs (Zhang et al. 2012). The proliferation of iPSC‐MSC_LV_^myo^ has been greatly suppressed (data not shown). Myocardin possibly downregulates the expression of the ether à go‐go 1 potassium channel and thus inhibits the proliferation of iPSC‐MSC_LV_^myo^.

With a conduction gap in an NRVM‐seeded MEA plate filled with desired cells, Pijnappels et al. (2006) measured the conduction velocities of BM‐MSCs and NRVMs to be 0.014 ± 0.004 m/sec and 0.168 ± 0.002 m/sec, respectively. Moreover, they have demonstrated that the electrical conduction of BM‐MSCs is caused by the expression of Cx43. Subsequently, they further showed that the forced myocardin expression induces the human ventricular scar fibroblasts to repair the conduction block of the NRVM field, possibly mediated by the upregulation of the expression of Cx40 and Cx45 (van Tuyn 2003). In contrast to these observations, our results showed that Cx40 and Cx45 expression, but not Cx43 were reduced upon myocardin transduction. While Cx40 and Cx45 are expressed in the embryonic heart, their expressions in the adult heart are mainly localized to pacemaker and conduction system. On the other hand, Cx43 is abundantly expressed in the myocardium. Therefore, downregulation of Cx40 and Cx45 after myocardin transduction may represent a transdifferentiation to a more mature ventricular phenotype (Verheule and Kaese 2013). Nevertheless, the conduction velocity of NRVMs cocultured iPSC‐MSC_AD_^myo^ was significantly faster than that of their GFP counterparts. Therefore, forced myocardin expression increased conduction velocity possibly because of its effects on the ion channel expression of iPSC‐MSCs rather than changes in connexins expression, in particular, by activating the expression of INa. TTX. Moreover, this increase in conduction velocity might account for the superior therapeutic potential of myocardin transduced BM‐MSCs than that of BM‐MSCs themselves in the mouse model of myocardial infarction (Grauss et al. 2008). These results suggest that similar to BM‐MSCs, iPSC‐MSCs are capable of conducting the electrical signals from NRVMs. More importantly, forced myocardin expression enhances the electrical conduction of iPSC‐MSCs.

The forced myocardin expression in human iPSC‐MSCs lead to partial transdifferentiation into cardiomyocytes and SMC phenotypes, modified the expression profile of the ion channels, and improved the electrical conduction velocity during coculturing with NRVM. The changes in the electrophysiological properties of iPSC‐MSC can potentially improve their electrical coupling with native cardiomyocytes after transplantation, thus reducing the risk of proarrhythymias. However, the therapeutic effect of the ectopic expression of myocardin in iPSC‐MSCs for cardiac repair and regeneration warrants further investigation in in vivo animal models of cardiac injuries such as myocardial infarction.

## Conflict of Interest

None declared.

## References

[b1] Amin A. S. Tan H. L. Wilde A. A. M. 2010 Cardiac ion channels in health and disease. Heart Rhythm; 7:117-126 19875343 10.1016/j.hrthm.2009.08.005

[b2] Borsani G. Rugarli E. I. Taglialatela M. Wong C. Ballabio A. 1995 Characterization of a human and murine gene (*clcn3*) sharing similarities to voltage‐gated chloride channels and to a yeast integral membrane protein. Genomics; 27:131-141 7665160 10.1006/geno.1995.1015

[b3] Chen J. F. Wang S. Wu Q. Cao D. Nguyen T. Chen Y. 2008 Myocardin marks the earliest cardiac gene expression and plays an important role in heart development. Anat. Rec. (Hoboken); 291:1200-1211 18780304 10.1002/ar.20756 PMC2694184

[b4] Deng X. L. Sun H. Y. Lau C. P. Li G. R. 2006 Properties of ion channels in rabbit mesenchymal stem cells from bone marrow. Biochem. Biophys. Res. Commun.; 348:301-309 16876113 10.1016/j.bbrc.2006.07.054

[b5] Du K. L. Ip H. S. Li J. Chen M. Dandre F. Yu W. 2003 Myocardin is a critical serum response factor cofactor in the transcriptional program regulating smooth muscle cell differentiation. Mol. Cell. Biol.; 23:2425-2437 12640126 10.1128/MCB.23.7.2425-2437.2003 PMC150745

[b6] Gaborit N. Le Bouter S. Szuts V. Varro A. Escande D. Nattel S. 2007 Regional and tissue specific transcript signatures of ion channel genes in the non‐diseased human heart. J. Physiol.; 582:675-693 17478540 10.1113/jphysiol.2006.126714 PMC2075332

[b7] Grauss R. W. van Tuyn J. Steendijk P. Winter E. M. Pijnappels D. A. Hogers B. 2008 Forced myocardin expression enhances the therapeutic effect of human mesenchymal stem cells after transplantation in ischemic mouse hearts. Stem Cells; 26:1083-1093 18203678 10.1634/stemcells.2007-0523

[b8] Hoofnagle M. H. Neppl R. L. Berzin E. L. Teg Pipes G. C. Olson E. N. Wamhoff B. W. 2011 Myocardin is differentially required for the development of smooth muscle cells and cardiomyocytes. Am. J. Physiol. Heart Circ. Physiol.; 300:H1707-H1721 21357509 10.1152/ajpheart.01192.2010 PMC3094091

[b9] Huang J. Min Lu M. Cheng L. Yuan L. J. Zhu X. Stout A. L. 2009 Myocardin is required for cardiomyocyte survival and maintenance of heart function. Proc. Natl. Acad. Sci. USA; 106:18734-18739 19850880 10.1073/pnas.0910749106 PMC2773995

[b10] Jin N. G. Kim J. K. Yang D. K. Cho S. J. Kim J. M. Koh E. J. 2003 Fundamental role of clc‐3 in volume‐sensitive Cl^−^ channel function and cell volume regulation in AGS cells. Am. J. Physiol. Gastrointest. Liver Physiol.; 285:G938-G948 12842831 10.1152/ajpgi.00470.2002

[b11] Lee Y. K. Ng K. M. Lai W. H. Chan Y. C. Lau Y. M. Lian Q. 2011 Calcium homeostasis in human induced pluripotent stem cell‐derived cardiomyocytes. Stem Cell Rev.; 7:976-986 21614516 10.1007/s12015-011-9273-3 PMC3226695

[b12] Li G. R. Feng J. Wang Z. Fermini B. Nattel S. 1995 Comparative mechanisms of 4‐aminopyridine‐resistant Ito in human and rabbit atrial myocytes. Am. J. Physiol.; 269:H463-H472 7653610 10.1152/ajpheart.1995.269.2.H463

[b13] Li G. R. Feng J. Yue L. Carrier M. 1998 Transmural heterogeneity of action potentials and Ito1 in myocytes isolated from the human right ventricle. Am. J. Physiol.; 275:H369-H377 9683422 10.1152/ajpheart.1998.275.2.H369

[b14] Li G. R. Sun H. Deng X. Lau C. P. 2005 Characterization of ionic currents in human mesenchymal stem cells from bone marrow. Stem Cells; 23:371-382 15749932 10.1634/stemcells.2004-0213

[b15] Li G. R. Sun H. Y. Chen J. B. Zhou Y. Tse H. F. Lau C. P. 2009 Characterization of multiple ion channels in cultured human cardiac fibroblasts. PLoS One; 4:e7307 19806193 10.1371/journal.pone.0007307 PMC2751830

[b16] Lian Q. Zhang Y. Zhang J. Zhang H. K. Wu X. Zhang Y. 2010 Functional mesenchymal stem cells derived from human induced pluripotent stem cells attenuate limb ischemia in mice. Circulation; 121:1113-1123 20176987 10.1161/CIRCULATIONAHA.109.898312

[b17] Pijnappels D. A. Schalij M. J. van Tuyn J. Ypey D. L. de Vries A. A. van der Wall E. E. 2006 Progressive increase in conduction velocity across human mesenchymal stem cells is mediated by enhanced electrical coupling. Cardiovasc. Res.; 72:282-291 16956599 10.1016/j.cardiores.2006.07.016

[b18] Siu C. W. Tse H. F. 2012 Cardiac regeneration: messages from caduceus. Lancet; 379:870-871 22336188 10.1016/S0140-6736(12)60236-0

[b19] Small E. M. Warkman A. S. Wang D. Z. Sutherland L. B. Olson E. N. Krieg P. A. 2005 Myocardin is sufficient and necessary for cardiac gene expression in xenopus. Development; 132:987-997 15673566 10.1242/dev.01684

[b20] Tao R. Lau C. P. Tse H. F. Li G. R. 2007 Functional ion channels in mouse bone marrow mesenchymal stem cells. Am. J. Physiol. Cell Physiol.; 293:1561-1567 10.1152/ajpcell.00240.2007 17699636

[b21] Torrado M. López E. Centeno A. Medrano C. Castro‐Beiras A. Mikhailov A. T. 2003 Myocardin mRNA is augmented in the failing myocardium: expression profiling in the porcine model and human dilated cardiomyopathy. J. Mol. Med.; 81:566-577 12920479 10.1007/s00109-003-0470-7

[b22] van Tuyn J. Pijnappels D. A. de Vries A. A. de Vries I. van der Velde‐van Dijke I. Knaän‐Shanzer S. 2003 Fibroblasts from human postmyocardial infarction scars acquire properties of cardiomyocytes after transduction with a recombinant myocardin gene. FASEB J.; 21:3369-3379 17579192 10.1096/fj.07-8211com

[b23] van Tuyn J. Knaän‐Shanzer S. van de Watering M. J. de Graaf M. van der Laarse A. Schalij M. J. 2005 Activation of cardiac and smooth muscle‐specific genes in primary human cells after forced expression of human myocardin. Cardiovasc. Res.; 67:245-255 15907818 10.1016/j.cardiores.2005.04.013

[b24] Verheule S. Kaese S. 2013 Connexin diversity in the heart: insights from transgenic mouse models. Front. Pharmacol.; 4:81 23818881 10.3389/fphar.2013.00081 PMC3694209

[b25] Wang Z. Wang D. Z. Teg Pipes G. C. Olson E. N. 2003 Myocardin is a master regulator of smooth muscle gene expression. Proc. Natl. Acad. Sci. USA; 100:7129-7134 12756293 10.1073/pnas.1232341100 PMC165841

[b26] Wang L. Chen L. Jacob T. J. C. 2000 The role of clc‐3 in volume‐activated chloride currents and volume regulation in bovine epithelial cells demonstrated by antisense inhibition. J. Physiol.; 524:63-75 10747184 10.1111/j.1469-7793.2000.t01-1-00063.x PMC2269844

[b27] Wang D. Chang P. S. Wang Z. Sutherland L. Richardson J. A. Small E. 2001 Activation of cardiac gene expression by myocardin, a transcriptional cofactor for serum response factor. Cell; 105:851-862 11439182 10.1016/s0092-8674(01)00404-4

[b28] Williams A. R. Hare J. M. 2011 Mesenchymal stem cells: biology, pathophysiology, translational findings, and therapeutic implications for cardiac disease. Circ. Res.; 109:923-940 21960725 10.1161/CIRCRESAHA.111.243147 PMC3604746

[b29] Xiong D. Heyman N. S. Airey J. Zhang M. Singer C. A. Rawat S. 2010 Cardiac‐specific, inducible clc‐3 gene deletion eliminates native volume‐sensitive chloride channels and produces myocardial hypertrophy in adult mice. J. Mol. Cell. Cardiol.; 48:211-219 19615374 10.1016/j.yjmcc.2009.07.003 PMC2879146

[b30] Yoshida T. Sinha S. Dandré F. Wamhoff B. R. Hoofnagle M. H. Kremer B. E. 2003 Myocardin is a key regulator of CArG‐dependent transcription of multiple smooth muscle marker genes. Circ. Res.; 92:856-864 12663482 10.1161/01.RES.0000068405.49081.09

[b31] Yoshida T. Kawai‐Kowase K. Owens G. K. 2004 Forced expression of myocardin is not sufficient for induction of smooth muscle differentiation in multipotential embryonic cells. Arterioscler. Thromb. Vasc. Biol.; 24:1596-1601 15231515 10.1161/01.ATV.0000137190.63214.c5

[b32] Zhang J. Chan Y. C. Ho J. C. Siu C. W. Lian Q. Tse H. F. 2012 Regulation of cell proliferation of human induced pluripotent stem cell‐derived mesenchymal stem cells via ether a go‐go 1 (hEAG1) potassium channel. Am. J. Physiol. Cell Physiol.; 303:C115-C125 22357737 10.1152/ajpcell.00326.2011

